# My Future: Psychodrama and Meditation to Improve Well-Being Through the Elaboration of Traumatic Loss Among Italian High School Students

**DOI:** 10.3389/fpsyg.2020.544661

**Published:** 2021-01-18

**Authors:** Ines Testoni, Lucia Ronconi, Gianmarco Biancalani, Andrea Zottino, Michael Alexander Wieser

**Affiliations:** ^1^Department of Philosophy, Sociology, Education and Applied Psychology (FISPPA), University of Padua, Padua, Italy; ^2^IT and Statistical Services, Multifunctional Pole of Psychology, University of Padua, Padua, Italy; ^3^Institute of Psychology, University of Klagenfurt, Klagenfurt, Austria

**Keywords:** death education, well-being, psychodrama, meditation, traumatic loss, suicide, high school students

## Abstract

This study was designed as an action research aimed to help students to elaborate their feelings of traumatic grief, due to a car accident and a suicide of two of their classmates, in an Italian high school. A death education project was realized in order to prevent the Werther effect. The intervention was based on psychodramatic techniques and meditation with Tibetan bells to encourage reflection on the suffering of traumatic loss, the sense of life, and their future. A total of 89 students from four classes (46 in the experimental group: two classes, 43 in the control groups: two classes) participated in the study, among which 82 (45 in the experimental group, 37 in the control group) completed the pre- and post-test survey. The intervention consisted of eight 2-h meetings, during which the themes of death and loss were dealt with through theoretical discussions, dramatization, and meditation. Two other classes which participated in the assessment as a control group did not attend the activities. The following instruments were used: Death Attitude Profile-Revised, which measures individual attitudes toward death; Psychological Well-being Scale, which measures a person’s psychological well-being; Resilience Scale for Adolescents, which measures the construct of resilience in adolescents; Self-Transcendence Scale, which measures self-transcendence; and Testoni Death Representation Scale, which measures the ontological representations of death. The results demonstrated that in the experimental group, there was a reduction in the fear of death and its avoidance, and that the students normalized the representation of death as something natural, thus improving their well-being. It is consequently possible to say that well-being is not simply the absence of suffering and worries, but rather, is rooted in the possibility of thinking of creative solutions to the trauma.

## Introduction

Avoidance of dialogs about death is common in contemporary Western societies (Testoni et al., 2013), and this can be a problem when it becomes necessary to elaborate a traumatic loss. Traumatic loss is defined as the condition resulting from sudden and violent loss, as it can result from an accident, a suicide, a murder, a disaster, or a war, that can lead to problematic grief elaborations (Testoni, 2015). In fact, in the past, religion and the social representation of death as a passage toward eternal life enabled individuals to face their and others’ mortality through rites illustrating shared symbolisms (Testoni et al., 2015). Such world views encompassing spiritual and religious strategies were the basis of education about the sense of life and projects about the future. However, over time, these ideas and practices became widely dismissed that nowadays, a substantial materialism characterizes the more or less conscious conviction of individuals that death is an absolute annihilation (Testoni et al., 2016). Since the representation of death as an annihilation is really more threatening than the one that indicated immortality, the contemporary tendency toward death denial produces many negative effects (Solomon et al., 2017). In our secularized culture, traditional religions have eroded to the point that finding meaning to existential questions about life encounters a number of difficulties, especially among adolescents and young adults who have to develop a valuable existential project for their future (Ronconi et al., 2009; Codato et al., 2011). Indeed, the denial of death and of spiritual contents can lead to a suppression of death-related thoughts and emotions, as described by the so-called black hole hypothesis^1^ (Testoni et al., 2019a), and this censorship could go forward as “alexithymia,” that is the tendency toward impoverished emotional experience and language expressing feelings (Bagby et al., 1994). Furthermore, the lack of a shared social language about death and dying has caused a systematic removal of experiences related to serious illnesses and mourning. In particular, this censorship may leave children and adolescents in a representational space, occupied by more or less arbitrary ideas that may produce disorientation in their existential reflection (Corr, 1995; Testoni et al., 2019b). Indeed, adolescents increasingly experience sudden and tragic deaths of their peers, usually from accidents, suicide, and homicide (Cupit and Meyer, 2014; Cupit and Kuchta, 2017). The unexpected deaths of young people may be severely traumatic if not elaborated on how it happens in a death-denying culture. Often, materialism, fantastic sensationalism, and horror characterize their exposure to the death-related content widely distributed in a variety of media venues (Testoni et al., 2016).

On the other hand, religious and spiritual practices have been demonstrated to improve well-being, reducing fear of death in both younger and older adults. Countless studies have demonstrated that religiousness and spirituality are related to higher levels of health and well-being (Park, 2007; Testoni et al., 2016). For example, Dechesne et al. (2003) demonstrated that when belief in an afterlife is boosted, the typical effects of death anxiety triggered by mortality salience are diminished as compared with when belief in the afterlife is discouraged.

Many authors affirm that the reopening of an authentic dialog on death and dying may be useful to promote a conscious reflection on existential themes involving concerns about loss and beliefs in the afterlife (Testoni et al., 2018a). Specifically, interventions of death education utilizing arts therapies and psychodrama have been shown to be capable of presenting an adequate elaboration of traumatic loss and mourning, thereby enhancing spirituality (Testoni et al., 2018b).

Indeed, psychodrama helps individuals interact with others, engaging in the possibility of developing their own spontaneity and creativity. According to Moreno (1953), these just-mentioned factors are the essential components of health and psychological well-being, as they represent the basis of resilience and of the authentic development of emotional skills. Its fundamental strategy allows participants to identify themselves in very difficult and complex situations, running in parallel with externalization of the inner suffering and its creative solution. Although some distinctions have to be made between the two approaches, both art therapies and psychodrama trigger positive processes of self-awareness capable of changing dysfunctional or ineffective behaviors and attitudes. Art therapies imply a more detached approach with inner feelings, as the art vehicle promotes projective mechanisms and a cold elaboration of the facts involved. Psychodrama, otherwise, is characterized by a more direct approach to one’s emotions and life’s experiences. The Death Education courses that utilize such instruments seem to really be able to promote the psychological well-being of adolescents and young adults (Testoni et al., 2019a, c).

This study wanted to analyze the effect of a project primarily aimed at restoring well-being through psychodrama with adolescents who underwent traumatic loss after the death of a companion in a car accident and after the suicide of a friend of hers who was also their classmate. The substantial finality was to prevent the risk of a Werther effect, by which sociologist David Phillips (1974) means the risk of an emulation of the act of suicide by those who have been exposed to the news of an already occurred suicide.

## Aims

The project “My future: the future meets the present without forgetting the past” was born to safeguard the psychological well-being of the students of a high school in North Italy, where two traumatic deaths occurred: the first was related to the death of a young girl who suffered a car accident and the second was because of the suicide of a friend of hers. The mentioned losses occurred 9 months prior to the implementation of this project and occurred within 2 days of each other. The two events were not correlated in any way except for the fact that the victims came from the same school institute. It was considered appropriate to make an intervention to work out these two events with the surviving classmates to restore their well-being quickly, thus stopping any possible Werther effect. The intervention intended to carry out a profound reflection on the issues of finitude, mortality, and spirituality, activating a reflection on what the prospects concerning the after-death might be and on the sense of life in facing death.

The study was aimed to assess the effect of the use of art therapy and psychodramatic techniques in helping people to overcome a loss and projecting the future in a serene way; in promoting the creation of a bond with the inner spiritual dimension; and in producing critical thoughts about death which would not undergo non-scientific opinions or stereotypes. The fundamental hypothesis of the research was that the experimental group experiencing the intervention would develop a better attitude toward death and reach higher levels of well-being, or at least that it would maintain such levels similar to those of the control group (students who had not experienced death education intervention).

## The Research

### Participants

The project involved four classes within a high school of Northern Italy (two experimental groups and two control groups). Among a comprehensive number of 89 students who filled out the survey at least once (46 in the experimental group, 43 in the control group), we have complete data on 82 (45 in the experimental group, 37 in the control group). Two classes made up of 45 students in total, with 27 females and 18 males aged 15 to 18 (M = 15.98, SD = 1.12), were part of the experimental group and participated in the intervention. These two particular classes were the ones frequented by the victims and in which they had formed the most intense bonds. The other two classes made up of 37 students in total, with 33 females and four males aged 15 to 18 (M = 16.14, SD = 1.00), were part of the control group and did not take part in the intervention. Being the participants in the experimental group classmates with the victims, they could be more linked to them than the participants of the control group. However, given the restricted school context (only about 150 students attend that high school in a small village of northern Italy), even those who were part of the control group knew the victims well. We cannot therefore exclude that the news of these two traumatic losses deeply shocked them, too. Although both experimental and control groups were mainly composed of females, the proportion of females was greater in the control group (Chi-square = 8.81, *df* = 1, *p* = 0.003). There were no age differences between the two groups (*t* = −0.66, *df* = 80, *p* = 0.509). All participants in the experimental group were asked by their headteacher for their consent to participate in this project and were left free to choose, as to let the experience be fully lived and transformative.

Participants provided written informed consent before participating in the study and parents were involved in the consent process. The study followed the APA Ethical Principles of Psychologists and Code of Conduct and the principles of the Declaration of Helsinki. It received research ethics approval from the Health Sciences and Science Research Ethics Committee of the University of Padova (reference: DE6F02E1BCE787AEB865991D730DEB3E).

### Activities in the Death Education Intervention

The intervention which the 44 students of the experimental group took part in was divided into eight meetings lasting 2 h each and structured as follows:

First meeting took place 3 days after the pre-test. A philosophy lesson was held concerning the relationship between time and existence, especially in relation to the philosophers of ancient Greece and the myths of modern times. In particular, in this part of the intervention, it was possible to explore the ontological representations of death that the students unconsciously theorize and practice. The aim of this first session was to introduce the theme of death to the participants, especially regarding the philosophical origin of some of the ideas that modern people share about the end of life. The philosophy lesson led to the possibility to analytically criticize what common sense firmly considers to be death.

Second meeting took place 2 days after the philosophy lesson. A meditation with Tibetan bells was realized. At the beginning of the session, the history of the Tibetan bells was introduced, then the researchers illustrated the basic principles of sound physics and how sound causes a series of emotional and spiritual consequences in humans. Then, the students carried out a meditation with Tibetan bells in a guided imagination activity: the students had to immerse themselves in the fantasy of finding a particular flower in the vastness of a large lawn, a flower that symbolizes their inner world, with its potential and resources. Finally, the participants were trained, and subsequently tried, to ring the Tibetan bells and by doing so to distinguish the various sounds.

The third meeting started a week after the second meeting. Some activities with psychodramatic techniques were carried out, utilizing the initial warm-up, letting participants express the expectations and ideas they had with regard to the experiment recently made. Also, they were asked to express their personal thoughts about both the philosophy lesson regarding the theme of death, and the experience of guided meditation with Tibetan bells. This first phase was followed by a practical activity. Participants were asked to walk freely around the room, then to touch one classmate on the shoulder and share a smile and a thought about that specific day with him/her. In one particularly significant experience, the class was divided into three subgroups which were asked to represent three scenes in “animated sculptures” mode. The assignment was to represent one person’s life in both its safe and uncertain aspects. The first group represented the swing game, the second staged an airplane flying through a storm, and the third represented a ship sailing on a rough sea. Finally, the three groups of students were given the opportunity to express their ideas on life and existence following the experience they had just lived. There was no protagonist-centered psychodrama, but dialogs were promoted by dividing the class in little pairs. This was done in order to let everyone share their personal considerations about the future and the continuation of life after the death of a loved one.

Fourth meeting took place a week after the previous session. Another psychodrama session was held, in which guided and active imagination work focused on the imagination of their future and of after-life was carried out, with the subsequent production of a drawing representing an important content (in particular a positive resource that would be useful in the future, in the symbolic form of an object) to share with others for each of the participants. In this session, every participant was invited to dress up and wear a gold cloak, impersonating the part of a Wise Man who reveals something unconventional and sage about the future. The two experiences with psychodrama offered students the opportunity to speak loudly and to express their thoughts and feelings to the girls involved in the incidents as if they were actually present. The purpose of these two psychodrama sessions was to activate the possibility of a dialog between the participants, who survived death, and the loved ones who went away, in order to let the former speak freely their feelings toward the losses they went through. Moreover, the last session offered the students a way to come into contact with their wise and inner self, in order to stimulate a bond with their spirituality and according to the aim of the Tibetan bells lessons.

Fifth meeting took place 10 days after the previous session. Another meditation session was held with Tibetan bells, in which each participant indicated four fundamental events on the timeline: their birth, the present moment, a moment in the past in which they experienced success, and a future event that is interpreted as a difficulty in achieving one’s goals and dreams. This was a guided-imagery session, scanned by the rhythmic sound of Tibetan bells. This way, the students were able to redesign and reinterpret their own future in new ways, guided by the awareness of the resources that the past offered them.

**FIGURE 1:**
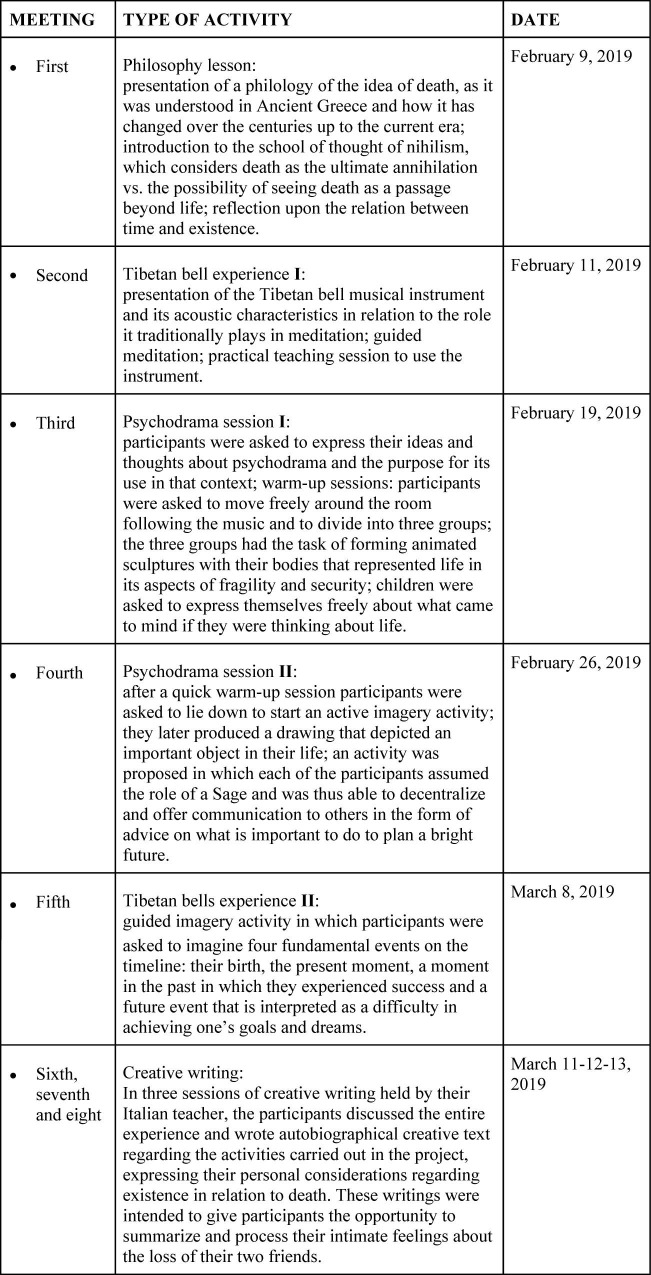
Structure of the activities.

Sixth, seventh, and eighth meetings took place respectively 3, 4, and 5 days after the last session with Tibetan bells. The students discussed the entire experience and wrote autobiographical creative text regarding the activities carried out in this project, expressing their personal considerations regarding existence in relation to death. These writings were intended to give participants the opportunity to summarize and process their intimate feelings about the loss of their two friends. No qualitative analysis procedure was meant to be proceeded with (Figure 1).

### Measurements

To evaluate the effectiveness of the intervention, 3 days pre- and 3 days post-intervention questionnaires were administered online. Each participant could access the online questionnaire, thanks to a code given by the researchers.

The questionnaires consisted of the following scales:

1. Death Attitude Profile-Revised (DAP-R; Wong et al., 1994) measures individual attitudes toward death. It is composed of 32 items on a 7-point Likert scale and is divided into five subscales: Fear of death (e.g., “Death is no doubt a grim experience”) (Cronbach’s alpha pre-test coefficient = 0.88 and post-test coefficient = 0.83), Death avoidance (e.g., “I avoid death thoughts at all costs”) (Cronbach’s alpha pre-test coefficient = 0.91 and post-test coefficient = 0.91), Neutral acceptance (e.g., “Death should be viewed as a natural, undeniable, and unavoidable event”) (Cronbach’s alpha pre-test coefficient = 0.68 and post-test coefficient = 0.67), Approach acceptance (e.g., “I believe I will be in heaven after I die”) (Cronbach’s alpha pre-test coefficient = 0.93 and post-test coefficient = 0.94), and Escape acceptance (e.g., “Death will bring an end to all my troubles”) (Cronbach’s alpha pre-test coefficient = 0.88 and post-test coefficient = 0.91).
2. Psychological Well-being Scale (PWBS; Ryff et al., 2007) measures the person’s psychological well-being. It is a 6-point Likert scale composed of 42 items and is divided into six subscales: Autonomy (e.g., “I am not afraid to voice my opinions, even when they are in opposition to the opinions of most people”) (Cronbach’s alpha pre-test coefficient = 0.75 and post-test coefficient = 0.79), Environmental mastery (e.g., “The demands of everyday life often get me down”) (Cronbach’s alpha pre-test coefficient = 0.79 and post-test coefficient = 0.82), Personal growth (e.g., “I do not enjoy being in new situations that require me to change my old familiar ways of doing things”) (Cronbach’s alpha pre-test coefficient = 0.64 and post-test coefficient = 0.64), Positive relations with others (e.g., “I often feel lonely because I have few close friends with whom to share my concerns”) (Cronbach’s alpha pre-test coefficient = 0.75 and post-test coefficient = 0.78), Purpose in life (e.g., “Some people wander aimlessly through life, but I am not one of them”) (Cronbach’s alpha pre-test coefficient = 0.68 and post-test coefficient = 0.70), and Self-acceptance (e.g., “In many ways, I feel disappointed about my achievements in life”) (Cronbach’s alpha pre-test coefficient = 0.83 and post-test coefficient = 0.87).
3. Resilience Scale for Adolescents (READ; Hjemdal et al., 2006) measures the construct of resilience in adolescents. It is composed of 22 items on a 5-point Likert scale and is divided into five subscales: Family cohesion (e.g., “I feel comfortable with my family”) (Cronbach’s alpha pre-test coefficient = 0.91 and post-test coefficient = 0.92), Personal competence (e.g., “I feel competent”) (Cronbach’s alpha pre-test coefficient = 0.83 and post-test coefficient = 0.82), Social competence (e.g., “I can easily make new friends”) (Cronbach’s alpha pre-test coefficient = 0.72 and post-test coefficient = 0.75), Social resources (e.g., “I always have somebody available when I need it”) (Cronbach’s alpha pre-test coefficient = 0.84 and post-test coefficient = 0.81), and Goal orientation (e.g., “I know how to achieve my goals”) (Cronbach’s alpha pre-test coefficient = 0.61 and post-test coefficient = 0.67). It correlates positively with mental health (Sense of Coherence Scale), dispositional optimism (Life Orientation Test), and self-esteem (Rosenberg Self-Esteem Scale) but negatively with depression and with scales indicating psychiatric symptoms (Hopkins Symptom Checklist, Automatic Thoughts Questionnaire, and Beck Hopelessness Scale).
4. Self-Transcendence Scale (STS; Reed, 2008) measures self-transcendence. The scale is composed of 15 items (e.g., “Finding meaning in my spiritual belief”) on a 4-point Likert scale, whose items measure the person’s ability to derive a sense of well-being through cognitive, creative, social, spiritual, and introspective resources. The scale showed a strong internal consistency (Cronbach’s alpha pre-test coefficient = 0.81 and post-test = 0.76). Self-transcendence is positively correlated with psychological well-being (Haugan et al., 2011). A high score in the STS is related to a higher self-transcendence.
5. Testoni Death Representation Scale (TDRS; Testoni et al., 2015) measures the ontological representations of death, which can be seen as a passage or annihilation. The scale is composed of six items (e.g., “Death is only a passage. After my death, I will continue to exist and remember the experiences of this life”), and agreement on each statement is evaluated on a 5-point Likert scale. The scale showed an acceptable internal consistency (Cronbach’s alpha pre-test coefficient = 0.79 and post-test = 0.79). A high score in the TDRS is related to an ontological representation of death seen as annihilation.

### Data Analysis

Our overall expectations included, for instance, that Death Education intervention would bring an improvement in the psychological well-being of the experimental group compared with the control group, and in the ability of the participants to positively face grief and elaborate loss. In order to examine those expectations, analysis of variance (ANOVA) was carried out on each measure with a within subjects’ factor “time” and a between subjects’ factor “group.” The time factor included a pre-test, which was filled out 3 days before the start of the experiment; and a post-test, which was filled out a week after the end of the experiment. The group factor included, as aforementioned, both an experimental and a control group. Partial eta-square was considered a measure of effect size for each ANOVA effect, in particular a value of 0.01 was interpreted as small effect, a value of 0.06 as medium effect, and a value over 0.14 as large effect. At the pre-test stage, the two groups were compared on all study variables using *t*-test and the change over time in each group was also examined by *t*-test. Coehen’s *d* was reported as measure of effect size for each *t*-test, in particular a value of 0.20 was interpreted as small effect, a value of 0.05 as medium effect, and a value over 0.08 as large effect. Correlations between all measures were evaluated by the Pearson correlation coefficient. In examining the correlations, we considered a classification of effect size: values from 0.10 to 0.25 indicate a small effect, values from 0.25 to 0.40 indicate a medium effect, and values over 0.40 indicate a large effect. Mediation and moderation analyses were carried out to evaluate the impact of DeEd intervention by global death attitude change on the students’ well-being and resilience at the post-test. The global attitude change was computed by adding the difference of the pre-test and the post-test for the two negative death attitude factors with a significant change over time, Fear of death and Death avoidance, and the difference of the post- and pre-test for the positive death attitude factor with a significant change over time, Neutral acceptance. All analyses were completed using IBM SPSS Statistics Version 25 and the macro-PROCESS for SPSS created by Preacher and Hayes for the mediation and moderation analyses (Hayes, 2018).

## Results

No significant difference between the two groups was found at the pre-test, except for one of the six subscales of PWBS—Environmental Mastery (*t* = 2.09, *df* = 80, *p* = 0.040, *d* = 0.46). The experimental group started with higher levels than the control group in this subscale (M = 3.95, SD = 0.82 vs. M = 3.55, SD = 0.91).

Correlations between measures at the pre-test are reported in Table 1. For the first subscale of DAP-R—Fear of death, we can observe only two significant correlations, both negative and with a small size, with the two subscales of PWBS—Environmental mastery and positive relations with others (*r* = −0.25 and *r* = −0.22, respectively). The two successive subscales of DAP-R—Death avoidance and neutral acceptance—show several positive significant correlations that are small to medium size with the subscales of PWBS (*r* range from 0.22 to 0.40 except for Death avoidance with Autonomy and for Neutral acceptance with Purpose in life, Environmental mastery, and Self-acceptance) and medium to large size with the subscales of READ (*r* range from 0.26 to 0.51 except for Neutral acceptance with Family cohesion and Goal orientation) and medium size with self-transcendence (*r* = 0.27 and *r* = 0.40, respectively). Considering the subscale of DAP-R—Approach acceptance, we can note only two significant correlations, a small positive correlation with Self-transcendence (*r* = 0.23) and a large negative correlation with Death as total annihilation of TDRS (*r* = −0.55). For the last subscale of DAP-R—Escape acceptance, we have several significant correlations, all negative and with a medium size, with the subscales of PWBS (*r* range from −0.25 to −0.30 except for Autonomy, Positive relations with others, and Personal growth) and with the subscale of READ—Goal orientation (*r* = −0.31). The six subscales of PWBS show significant positive correlations, all with a medium to large size, with all subscales of READ and with Self-transcendence (*r* range from 0.26 to 0.74, except for Autonomy with social resources and for Personal growth and Family cohesion) and significant negative correlations, with a small size, with Death as total annihilation of TDRS (*r* range from −0.22 to −0.26 except for Autonomy, Environmental mastery, and Personal growth). The five subscales of READ show significant large positive correlations with Self-transcendence (*r* range from 0.45 to 0.60). Only the subscale of READ—Social resources shows a significant small negative correlation with Death as total annihilation of TDRS (*r* = −0.22). Finally, there is a medium negative correlation between Self-transcendence and Death as total annihilation of TDRS (*r* = −0.27).

**TABLE 1:** Correlations between all measures at the pre-test.

Measures	1	2	3	4	5	6	7	8	9	10	11	12	13	14	15	16	17
1. Fear of death																	
2. Death avoidance	0.36**																
3. Neutral acceptance	−0.50***	–0.17															
4. Approach acceptance	0.15	0.22	0.07														
5. Escape acceptance	–0.20	−0.27*	0.19	0.29**													
6. Autonomy	–0.14	0.15	0.22*	0.18	–0.10												
7. Environmental mastery	−0.25*	0.32**	0.16	0.07	−0.28*	0.45***											
8. Personal growth	–0.11	0.26*	0.28*	0.13	–0.21	0.41***	0.42***										
9. Positive relations with others	−0.22*	0.23*	0.40***	0.14	–0.13	0.32**	0.56***	0.48***									
10. Purpose in life	0.06	0.34**	0.02	0.10	−0.25*	0.41***	0.60***	0.37**	0.31**								
11. Self-acceptance	–0.10	0.40***	0.21	0.19	−0.25*	0.51***	0.79***	0.42***	0.64***	0.59***							
12. Family cohesion	0.15	0.51***	–0.01	0.14	−0.30**	0.28*	0.49***	0.22*	0.36**	0.44***	0.56***						
13. Social competence	–0.07	0.30**	0.26*	0.08	–0.07	0.48***	0.48***	0.44***	0.61***	0.27*	0.49***	0.40***					
14. Personal competence	–0.19	0.39***	0.29**	0.21	–0.14	0.47***	0.66***	0.44***	0.60***	0.38***	0.74***	0.37**	0.58***				
15. Social resources	–0.08	0.40***	0.28*	0.11	–0.22	0.22*	0.48***	0.29**	0.54***	0.26*	0.60***	0.72***	0.40***	0.48***			
16. Goal orientation	–0.07	0.35**	0.18	0.03	−0.31**	0.43***	0.59***	0.37**	0.50***	0.48***	0.59***	0.44***	0.44***	0.57***	0.49***		
17. STS total score	–0.14	0.27*	0.40***	0.23*	–0.15	0.47***	0.56***	0.46***	0.52***	0.36**	0.63***	0.45***	0.45***	0.60***	0.51***	0.47***	
18. TDRS total score	0.08	–0.14	–0.15	−0.55***	–0.07	–0.21	–0.12	–0.15	−0.26*	−0.22*	−0.25*	–0.19	–0.07	–0.20	−0.22*	–0.10	−0.27*

*p < 0.05; **p < 0.01; ***p < 0.001.

The ANOVA results (Table 2) show a significant interaction time × group only for three subscales of DAP_R—Fear of death, Death avoidance, and Neutral acceptance. All interaction effects show a medium to large effect size. In particular, we can observe a significant decrement with a large effect size of the Fear and of the avoidance of death from pre- to post-test in the experimental group (*t* = 5.18, *p* < 0.001, *d* = 1.16, and *t* = 3.81, *p* < 0.001, *d* = 0.85, respectively) and no significant variation in the control group (*t* = 1.12, *p* = 0.265, *d* = 0.25, and *t* = −0.54, *p* = 0.590, *d* = −0.12, respectively). On the contrary, we can observe a significant increment with a medium to large effect size of Neutral acceptance of death from pre- to post-test in the experimental group (*t* = −3.19, *p* = 0.002, *d* = −0.71) and no significant variation in the control group (*t* = 0.17, *p* = 0.869, *d* = 0.04). Moreover, there is a significant main effect of time for the three subscales of DAP_R—Fear of death, Death avoidance, and Neutral acceptance—and one subscale of PWBS—Positive relations with others. Students showed decreased scores between pre- and post-test in all these measures except for Neutral acceptance of death, where their scores increased. Finally, there is a significant main effect of the group for the first subscale of DAP-R—Fear of death, and one subscale of PWBS—Environmental mastery. In fact, the experimental group showed lower levels of Fear and higher levels of Environmental mastery than the control group. There are no significant effects in the READ, STS, and TDRS.

**TABLE 2:** Descriptive statistics of all study variables by time for each group with ANOVA results.

Measures	Experimental group				Control group				ANOVA results^*a*^					
	Pre-test		Post-test		Pre-test		Post-test		Group		Time		Time × Group	
	M	SD	M	SD	M	SD	M	SD	F	Partial eta-square	*F*	Partial eta-square	*F*	Partial eta-square
***Death Attitude Profile-Revised (DAP-R)***														
Fear of death	4.35	1.52	3.67	1.64	4.88	1.25	4.72	1.32	6.66*	0.08	18.59***	0.19	7.02*	0.08
Death avoidance	4.02	1.67	3.30	1.60	3.94	1.67	4.05	1.51	1.05	0.01	4.66*	0.06	8.77**	0.10
Neutral acceptance	5.09	1.35	5.47	1.08	5.12	0.87	5.10	1.02	0.54	0.01	4.07*	0.05	5.12*	0.06
Approach acceptance	3.06	1.30	3.04	1.37	3.48	1.33	3.61	1.44	2.90	0.04	0.40	0.01	0.82	0.01
Escape acceptance	3.12	1.38	3.25	1.59	3.46	1.59	3.18	1.47	2.75	0.00	0.40	0.01	2.75	0.03
***Psychological Well-being Scale (PWBS)***														
Autonomy	4.13	0.88	4.16	0.86	4.00	0.79	4.05	0.88	0.42	0.01	0.37	0.01	0.01	0.00
Environmental mastery	3.95	0.82	3.93	0.89	3.55	0.91	3.53	1.03	4.34*	0.05	0.13	0.00	0.00	0.00
Personal growth	4.71	0.69	4.71	0.65	4.78	0.62	4.72	0.68	0.10	0.00	0.29	0.00	0.29	0.00
Positive relations with others	4.45	0.82	4.38	0.80	4.41	0.91	4.21	0.98	0.35	0.00	4.58*	0.05	1.20	0.02
Purpose in life	3.93	0.89	3.93	0.84	3.76	0.92	3.96	0.90	0.13	0.00	2.09	0.03	2.09	0.03
Self-acceptance	3.89	1.01	3.92	1.07	3.46	1.03	3.51	1.14	3.38	0.04	0.34	0.00	0.03	0.00
***Resilience Scale for Adolescents (READ)***														
Family cohesion	3.64	0.92	3.54	0.95	3.55	1.06	3.63	1.09	0.00	0.00	0.01	0.00	1.81	0.02
Social competence	3.51	0.90	3.51	0.77	3.36	0.84	3.30	0.96	0.93	0.01	0.30	0.00	0.30	0.00
Personal competence	3.42	0.91	3.43	0.84	3.20	0.73	3.17	0.87	1.98	0.02	0.01	0.00	0.08	0.00
Social resources	3.88	0.93	3.82	0.88	3.86	0.91	3.76	0.91	0.04	0.00	1.44	0.02	0.04	0.00
Goal orientation	3.86	0.71	3.93	0.73	3.87	0.69	3.78	0.77	0.21	0.00	0.01	0.00	1.52	0.02
***Self-Trascendence Scale (STS)***														
STS total score	3.15	0.48	3.22	0.42	3.06	0.31	3.09	0.31	1.92	0.02	2.19	0.03	0.33	0.00
***Testoni Death Representation Scale (TDRS)***														
TDRS total score	3.35	0.78	3.20	0.80	3.09	0.87	3.09	0.82	1.23	0.02	0.91	0.01	0.91	0.01

^a^Degrees of freedom are 1 and 80 for all F test.*p < 0.05; **p < 0.01; ***p < 0.001.

Finally, mediation analysis to estimate the indirect effect of death education intervention through global death attitude change on the students’ well-being and resilience at the post-test was performed. Results show a significant positive indirect effect for the subscale Autonomy of PWBS and for the subscale Goals of READ (Table 3). Moreover, we carried out a moderation analysis to estimate the impact of negative death attitude reduction on students’ well-being and resilience at the post-test in the experimental and in the control group. In each model, we included the same measure at the pre-test as covariate, the global death attitude changes as focal predictor, and the death education intervention (coded as dummy variable: 1 = Yes and 0 = No) as moderator. A significant moderating effect of death education was found only for some subscales of PWBS but none of the subscales of READ. In Table 4, we report the results for all subscales of PWBS. We can observe a significant interaction of global death attitude change by death education intervention for Environmental mastery and Purpose in life and a close to significant interaction for Autonomy. The conditional effects of global death attitude change by death education intervention show that in the experimental group, negative death attitude reduction contributes to increased well-being at the post-test (B = 0.12, SE = 0.04, *t* = 2.97, *p* = 0.004 for Autonomy; B = 0.05, SE = 0.04, *t* = 1.34, *p* = 0.185 for Environmental mastery; and B = 0.04, SE = 0.04, *t* = 1.02, *p* = 0.313 for Purpose in life) but not in the control group (B = 0.02, SE = 0.04, *t* = 0.59, *p* = 0.556 for Autonomy; B = −0.06, SE = 0.04, *t* = −1.56, *p* = 0.122 for Environmental mastery; and B = −0.10, SE = 0.04, *t* = −2.59, *p* = 0.011 for Purpose in life).

**TABLE 3:** Mediation effect of global death attitude change on death education intervention impact on student’s well-being and resilience at the post-test.

Dependent variable	Indirect effect of death education intervention (by global death attitude change)				
	B	SE	*t*	95% Confidence interval—bootstrap estimation	
				Lower level	Upper level
***PWBS subscales***					
Autonomy	0.12	0.06	2.00*	0.02	0.24
Environmental mastery	–0.01	0.04	–0.25	–0.10	0.06
Personal growth	0.01	0.05	0.20	–0.09	0.11
Positive relations with others	0.06	0.06	1.00	–0.04	0.20
Purpose in life	–0.06	0.06	–1.00	–0.18	0.05
Self-acceptance	0.01	0.05	0.20	–0.09	0.11
***READ subscales***					
Family cohesion	–0.04	0.06	–0.67	–0.16	0.06
Social competence	0.08	0.06	1.33	–0.02	0.21
Personal competence	0.06	0.05	1.20	–0.05	0.15
Social resources	–0.02	0.05	–0.40	–0.14	0.08
Goal orientation	0.14	0.06	0.233*	0.02	0.25

*p < 0.05.

**TABLE 4:** Moderation effect of death education intervention on global death attitude change impact to student’s well-being at the post-test.

	Autonomy			Environmental mastery			Personal growth			Positive relations with others			Purpose in life			Self-acceptance		
						
	B	SE	*t*	B	SE	*t*	B	SE	*t*	B	SE	*t*	B	SE	*t*	B	SE	*t*
Measure at the pre-test^1^	0.80	0.07	11.18***	0.93	0.07	13.68***	0.69	0.08	8.43***	0.082	0.07	12.08***	0.76	0.07	10.86***	0.91	0.07	13.46***
Global death attitude change	0.02	0.04	0.06	–0.06	0.04	−1.56	–0.02	0.03	−0.72	0.07	0.04	1.88∼	–0.10	0.04	−2.59*	−0.03	0.04	−0.64
Death education intervention (1 = Yes; 0 = No)	–0.2	0.14	−1.45	–0.06	0.14	−0.47	–0.03	0.12	−0.28	0.15	0.14	1.14	–0.23	0.14	−1.66	−0.06	0.16	−0.40
Interaction (global death attitude change by Death education intervention)	0.1	0.06	1.75∼	0.11	0.05	2.05*	0.07	0.05	1.32	–0.08	0.05	−1.41	0.14	0.06	2.58*	0.07	0.06	1.16

^1^The corresponding variable at the pre-test for each dependent variable is included.∼p < 0.10; *p < 0.05; **p < 0.01; ***p < 0.001.

## Discussion

Regarding the results of the correlations between the instruments, the dimensions of personal well-being and resilience are positively correlated with each other. This shows how personal well-being increases, and vice versa, as resilience increases. It is also noted that as psychological well-being increases, transcendence also increases, highlighting an influence between these two constructs. Furthermore, as a high availability of positive relationships with others increases, a representation of death as annihilation decreases. For self-transcendence, this is correlated with resilience in a directly proportional way, so people who are able to expand personal boundaries in more ways are also more capable of restoring their psychological balance after having faced and passed a traumatic event or a period of difficulty. Finally, the correlations show that a greater vision of death as annihilation leads to a reduction of cohesion within the family and a decrease in the availability of social resources.

Both decrease in the fear of death and decrease in the avoidance of death were observed in the experimental group, making it the main result obtained in our study. In fact, an intervention of Death Education at school, which adequately and consciously deals with the theme of death and the elaboration of a mourning seems to reduce terror and anguish toward the end of life. Furthermore, the avoidance of death is what characterizes the communal and communicative dynamics that cover Western culture, which makes death a pornography, a taboo of which nothing can be said (Gorer, 1965). In this case, we observed that, in addition to the objectives and constructs investigated, the psychodrama intervention aided the participants in enhancing their ability to discuss and communicate intimately and in depth their own feelings and experiences regarding the losses that occurred during each person’s lifetime.

Another important result was the increase in the neutral acceptance of death, that is, of perceiving death and forgiveness that derive from it as a natural event, which correlates negatively with depression and positively with psychological well-being (Wong et al., 1994). Neutral acceptance of death is significantly and positively correlated with self-transcendence, indicating that the ability to transcend one’s self allows one to observe death and accept it in a serene way, as observed by Tacòn (2018) and Schultz and Arnau (2017). Nevertheless, it has to be noticed that self-transcendence did not give significant results in the experimental group. We consider that to be due to the insufficient amount of time for observing developments and change. Also, the Death Education project would have required a longer duration.

The psychological well-being of the experimental group remains stable, and this is fundamental as it highlights that talking about death and taking part in experiences regarding this theme does not affect their psychological balance. An increase in this variable would have been desirable, but the fact of not having decreased one’s personal well-being is to be considered a good result: this is in fact very likely to happen when the subject of death is approached in an inappropriate way.

The results of the mediation analysis show how the path of death education, through a positive change in the vision of death, or a decrease in fear and avoidance of death on one hand and an increase in neutral acceptance on the other, seems to favor personal well-being and resilience. In particular, it increases the ability to be self-confident and to resist the social pressures that lead to confirming one’s thinking to that of others and regulating one’s behavior from within (Ruini et al., 2003). In addition, the importance of visualizing the future and having essential objectives to discover the meaning of life (Vanistendael, 2005) and the perception of one’s ability to reach them also increase (Theis, 2003; Vanistendael, 2003; Saavedra and Villalta, 2008).

The results of the moderation analysis show how a positive change in the vision of death seems to bring an increase in well-being in those who participated in the Death Education path. In fact, these participants have a greater sense of domination and competence in controlling the surrounding environment, increasing their degree of management in a wide range of activities while also managing to make the surrounding environment the most compliant with their needs. In addition, with the path of death education, they show that they have developed the beliefs that give meaning to their lives, providing a direction to their personal path, thus allowing them to attribute an important meaning to both the past and the present. Finally, the participants increased almost significantly their ability to be self-confident and independent and to regulate their behavior from within by evaluating themselves through personal standards (Ruini et al., 2003).

## Conclusion

This study shows that a Death Education project which involves both a creative (such as psychodrama) and a meditative (such as the use of Tibetan bells) method, can be effective in reducing the fear and avoidance of death even when facing fatal accidents and suicide among adolescents. By measuring the effects of this project, it is in fact possible to confirm what other projects have already shown, namely, it is not the removal of death that helps in grief management. In this sense, the scenic action of psychodramatic techniques adapted to the school context, together with a reflection about what death means for philosophy contra common sense and with the possibility to activate one’s inner spiritual dimension, it all helps express, elaborate, and overcome a sudden and unexpected loss. This is made possible thanks to the opening of an extensive reflection about the future, the meaning of life despite its limits, and the pain that often afflicts us. The appreciation of the spiritual sphere also proves to be very important for the elaboration of the loss and reflection on the future. To offer young people a perspective structurally different than the merely materialistic ones that characterize contemporary culture is a value and an aim which we strongly endorse. We therefore can say that giving someone the chance to tell and to elaborate feelings and thoughts on loss decreases the nihilist fear of death as a point of no return. Using strategies and techniques like philosophy-centered lessons, psychodrama, use of Tibetan bells, and creative writing, as it happened in this action-research project, helps participants in freeing themselves from anguish and to accept death as a stage of life. It has to be noticed that techniques such as the psychodrama and the Tibetan bells helped participants to enter in contact with their inner spiritual dimension, although the post-test survey could not notice it. We hypothesize that such a result can be related to the nature of spirituality itself, which is not an immediate conquest but instead it requires to be cultivated and grown in times that cannot be adequately recorded by post-test questionnaires.

## Limitations of the Study

The main limitation of this study is that the measurements were made on previously established (school classes) and non-randomized groups. Moreover, the number of participants is relatively small. Furthermore, once this intervention with the experimental group was concluded, it would have been appropriate to offer this same intervention to the control group to allow them to elaborate the event of the deaths of their two schoolmates.

## Data Availability Statement

The raw data supporting the conclusions of this article will be made available by the authors, without undue reservation.

## Ethics Statement

The studies involving human participants were reviewed and approved by Ethics Committee of the University of Padova (DE6F02E1BCE787AEB865991D730DEB3E). Written informed consent to participate in this study was provided by the participants’ legal guardian/next of kin.

## Author Contributions

IT is the scientific director of the present research project; she therefore elaborated the study design, and supervised its implementation. She also contributed in the interpretation of the data and the recognition of the main results, supervised the elaboration of the article, and wrote its final version. LR is responsible for analyzing qualitative the data. She also dealt with the choice of pre- and post-intervention measurement tests and she actively contributed to the writing of the results section. GB collaborated in the elaboration of the study design, in the choice of the psychological constructs to be measured, and in the data collection on the online platform. He also contributed to the discussion of the results. AZ has been involved in research on the theoretical state of the art on literature and has collaborated in the implementation of the intervention. MW offered important suggestions for the enhancement of the arts therapy intervention in the high school and participated in the discussion of the results. All authors contributed to the article and approved the submitted version.

## Conflict of Interest

The authors declare that the research was conducted in the absence of any commercial or financial relationships that could be construed as a potential conflict of interest.

## Footnotes

1 The “Black Hole hypothesis” states that “the tendency toward impoverished emotions (Bagby et al., 1994) or alexithymia could be related to a systematic cultural inhibition of dialog on death and dying” (Testoni et al., 2019a).
